# *In Vitro* Assembly Kinetics of Cytoplasmic Intermediate Filaments: A Correlative Monte Carlo Simulation Study

**DOI:** 10.1371/journal.pone.0157451

**Published:** 2016-06-15

**Authors:** Norbert Mücke, Stefan Winheim, Holger Merlitz, Jan Buchholz, Jörg Langowski, Harald Herrmann

**Affiliations:** 1 Division Biophysics of Macromolecules, German Cancer Research Center (DKFZ), Heidelberg, Germany; 2 Department of Physics and ITPA, Xiamen University, Xiamen, China; 3 Institute of Neuropathology, University Hospital Erlangen, Erlangen, Germany; Dalhousie University, CANADA

## Abstract

Intermediate filament (IF) elongation proceeds via full-width “mini-filaments”, referred to as “unit-length” filaments (ULFs), which instantaneously form by lateral association of extended coiled-coil complexes after assembly is initiated. In a comparatively much slower process, ULFs longitudinally interact end-to-end with other ULFs to form short filaments, which further anneal with ULFs and with each other to increasingly longer filaments. This assembly concept was derived from time-lapse electron and atomic force microscopy data. We previously have quantitatively verified this concept through the generation of time-dependent filament length-profiles and an analytical model that describes assembly kinetics well for about the first ten minutes. In this time frame, filaments are shorter than one persistence length, i.e. ~1 μm, and thus filaments were treated as stiff rods associating via their ends. However, when filaments grow several μm in length over hours, their flexibility becomes a significant factor for the kinetics of the longitudinal annealing process. Incorporating now additional filament length distributions that we have recorded after extended assembly times by total internal reflection fluorescence microscopy (TIRFM), we developed a Monte Carlo simulation procedure that accurately describes the underlying assembly kinetics for large time scales.

## Introduction

Intermediate filament (IF) proteins are important constituents of the metazoan cytoskeleton as well as the nuclear matrix [1]. In man, 70 genes code for distinct tissue specific IF proteins [2,3]. All IF proteins are fibrous, i.e. they are extended molecules exhibiting a tripartite structure with a central, mostly α-helical domain forming the “rod”. In cytoplasmic IF proteins it exhibits a length of approximately 46 nm. The rod domain is flanked by unstructured or intrinsically disordered non-α-helical amino-terminal “head” and carboxy-terminal “tail” domains; in both cases their length varies extensively when different IF proteins are compared [4,5]. A central issue for understanding the function of IF systems is to know their mechanism of assembly.

The occurrence of assembly intermediates and the kinetics of filament formation were investigated early on by different techniques such as light scattering [6], viscosity measurements [7] as well as by electron microscopy (EM) [8,9]. In continuation of these studies, advanced techniques such as quantitative high resolution EM in parallel with atomic force microscopy (AFM), light microscopy of fluorescently labeled subunits including total internal reflection fluorescence microscopy (TIRFM), small angle X-ray scattering (SAXS) and microfluidic techniques in combination with time-resolved SAXS were applied [10–14].

The assembly of the cytoplasmic IF protein vimentin proceeds in three phases [15]. A tetrameric complex, consisting of two parallel coiled coils oriented in a half-staggered, antiparallel conformation, represents the smallest molecular unit that can be stably kept in solution under non-denaturing conditions *in vitro*. Notably, the presence of tetrameric complexes in cultured cells, though in minute amounts, has been established by sucrose-density centrifugation of high-speed supernatants from cultured cell extracts [16]. The tetramers are 60 nm long and proper homogenous complexes are obtained by sedimentation velocity studies at low ionic strength [8,17–19]. Recently, a molecular model of the tetramer at atomic resolution was presented highlighting some details of the coiled-coil interaction [20–22].

For kinetic analyses, assembly is initiated by a sudden rise of the ionic strength. This procedure results in the formation of *bona fide* IFs as revealed by transmission electron microscopy of negatively stained specimen, by scanning transmission electron microscopy (STEM) of unstained samples and by cryo-electron microscopy of vitrified specimens [23].

In the first phase of assembly, full-width unit-length filaments (ULF) of 60 nm length are formed within seconds by the lateral association of tetramers [15]. In the second phase, ULFs longitudinally anneal with each other and newly formed filaments, and thereby filaments grow with a distinct speed yielding filaments with increasing numbers of ULF segments in a time-dependent mode [12]. After 20 min of assembly under “standard conditions”, filaments exhibit a broad length-distribution with individual IFs ranging from 60 nm to more than 2.4 μm, i.e. from one ULF to filaments consisting of up to 56 ULF-subunits [24]. By increasing the protein concentration and the ionic strength, assembly kinetics speed up dramatically. However, single filament length determination is impossible under these conditions due to the heavy association of single filaments into thick bundles [25]. Moreover, the speed of assembly differs significantly when IF proteins from distinct assembly groups such as keratins, vimentin and desmin are compared [26].

Both the lateral association of extended molecules to short minifilaments and the end-to-end annealing as a mechanism for filament elongation are not unique for IF proteins. Actually, the lateral association of antiparallel coiled-coil molecules to minifilaments has been described for myosin [27] and the end-to-end annealing of short filaments as a means of filament elongation, has originally also been observed for the polymerization of actin filament from monomers [28], and more clearly, with short fluorescently labeled actin filaments that were obtained by shearing of long filaments [29].

The actual length of filaments measured by EM and AFM is influenced by three interdependent effects: i) filaments can elongate during the deposition process; ii) the diffusion coefficient varies with filament size and hence short filaments may reach the surface faster than long ones; iii) long filaments can interact with the surface via one of the two ends even if their center of mass is far away and therefore can be trapped preferentially. We demonstrated that for filaments of up to 1 μm the deposition time does not influence the filament length distribution measurements [12]. In contrast, for filaments longer than 1 μm the three above-mentioned points may become relevant. Therefore we developed a protocol for TIRFM of fluorescently labeled vimentin such that filaments could rapidly attach to the support: first, we stopped the assembly by diluting the sample several hundred-fold, then filament deposition was done in a confined space with a reaction chamber height of 16 μm.

For the quantitative description of assembly, we had previously established an analytical model based on rod-like linear elements elongating by end-on annealing [30]. To analyze whether the model would also hold for assembly times, when the filaments are several times the persistence length, we now measured filaments after two and four hours of assembly, when they are several micrometers long, and applied the analytical model to these data [31]. However, in this case the analytical model based on rod-like linear elements failed. For long incubation times, Portet extended the model by including flexibility in an approximate way, by estimating the diffusion-limited reaction rate from the Smoluchowski equation on the basis the average dimension of a filament of given flexibility. This necessitated the use of different limiting cases for long (globular) and short (rodlike) molecules. Because of these apparent difficulties, we chose a different approach and developed a numerical Monte Carlo procedure for the kinetics of filament assembly that included the flexibility of IFs as a central parameter. This model now allows us to describe vimentin filament formation at high precision over a wide range of assembly times. In a further step, we compared the assembly kinetics of vimentin with those of desmin and keratin K8/K18. We reveal that filament assembly of all three types of cytoplasmic IF proteins can be described by the same principal assembly parameters indicating they share the same molecular mechanism of assembly.

## Materials and Methods

### Protein chemical procedures

Recombinant human vimentin, desmin and keratins K8 and K18 were purified from transformed bacteria, TG1 (Amersham, Braunschweig, Germany), (as described [8,32,33]. Alexa 488-labeled vimentin was prepared as described [14]. For assembly, labeled vimentin was mixed with unlabeled vimentin (1: 9) in 8 M urea.

### Filament assembly

Proteins were renatured from 8 M urea by dialysis into 2 mM sodium phosphate, pH 7.5 (*phosphate buffer*), or 5 mM Tris-HCl, pH 8.4 (*Tris-buffer*). Assembly mode 1 (NaP_i_): The assembly of both vimentin and desmin was started at 37°C by addition of an equal volume of *phosphate buffer* containing 200 mM potassium chloride to yield 2 mM NaPi, pH 7.5, 100 mM potassium chloride (*assembly buffer*). Assembly was stopped either after 10 min or after 2 and 4 hours, respectively, by diluting the sample 1:250 with *assembly buffer* followed by processing for EM and AFM [34]. For TIRFM, 10 to 50 μl of sample were applied onto a glass support and immediately covered with a glass coverslip (25 mm x 25 mm) resulting in a chamber height of 16 μm. By this procedure a flow of the filaments during the adsorption process is not prevented and therefore mechanical properties like the persistence length were not determined. After a few minutes all filaments were adsorbed onto the untreated glass support with no free particles or filaments observed in the free pool and imaging was started immediately. Assembly mode 2 (Tris-NaCl): Desmin was renatured into 5 mM Tris-HCl (pH 8.4) and assembly was started by addition of an equal volume of 45 mM Tris-HCl (pH 7.0) buffer containing 100 mM sodium chloride. Assembly mode 3 (Tris-HCl): The keratin pair was renatured into 2 mM Tris-HCl, pH 9.0, and assembly was initiated by addition of an equal volume of 18 mM Tris-HCl, pH 7.0. The final protein concentration was 0.1 g/l for vimentin, 0.02 or 0.1 g/l for desmin, and 0.0025 g/l for keratins.

### Image acquisition and analysis

EM analysis of vimentin and keratin IFs and AFM analysis of vimentin and desmin were performed exactly as described previously [12,30]. TIRFM of fluorescently labeled IFs was done essentially as described [14]. All images were processed using ImageJ v.1.42q (NIH). The backbones of the filaments were traced manually using a Wacom LCD tablet (Cintiq21UX). To reduce errors due to the pixelization of the images, individual filaments contours were smoothed by using the weight average of five contiguous XY-coordinates centered about a given XY-coordinate [34]. The filament lengths (*L*) were converted into number of ULFs (*i*) by [(*L*—60) / 42.7] + 1 = *i* [30]. The length of a single ULF is around 60 nm and if two ULFs or filaments fuse, the corresponding ends interdigitate by approximately 17 nm.

### The Monte Carlo simulation procedure

To simulate the complex filament assembly we developed a Monte Carlo (MC) simulation procedure in continuum space that allows us to introduce filament flexibility as a main structural parameter. This procedure is sufficiently fast to cover the wide range of time scales needed to form long fibers in a diffusion-driven process and the large range of length scales from the length of a single ULF up to several μm long filaments. The fibers were modeled using a simple bead-bond model, in which, similar to the bond fluctuation method [35], the bond lengths are allowed to vary within a certain interval. This setup yields a high acceptance rate and reasonably short simulation times.

A single ULF was made of four consecutive spherical beads of *d* = 11 nm diameter, serving as our standard length unit. The distance between two neighboring beads was allowed to vary between 0.8 and 1.2 *d* (bead diameter). This choice of the acceptance interval was the result of a compromise between a suitable acceptance rate, and, at the same time, to keep the average bond length close to unity (1.026 *d* in this case). The latter condition prevents an unphysical variation of the contour-length of the filament.

Torsion angles were entirely free, while the bond angles were allowed to fluctuate freely within a solid angle α. In this way, expensive evaluations of bending potentials and Boltzmann probabilities were avoided and conformations were rejected or accepted by purely geometrical criteria. During each MC time step (sweep) every single bead was moved, its new position checked for violations of the bond-length or bond-angle condition, then accepted or rejected. During each trial-move, the selected bead was displaced by a vector of random orientation and a magnitude that was uniformly sampled within the interval [0, 0.1] (in units of the bead-size), leading to an average acceptance rate of about 2/3 of the attempted moves. This acceptance rate implies that the numerical overhead of rejected trial-moves remains tolerable, while still allowing for reasonably fast conformation changes. The simulation box had fully periodic boundaries to prevent finite size effects through filament interactions with solid walls. Two filaments were allowed to assemble always if their ends approached each other below a distance of the maximum bond length, i.e., 1.2 *d*. To conserve the mechanical property of the newly formed filament, the reaction solid angle needs to be in the same range as the bond angle.

For the filaments that we investigate, i.e. with a persistence length of *l*_p_ and a contour length $l_{c}<l_{p}^{3}/d^{2}$, excluded volume effects do not influence the chain statistics significantly [36]. Therefore the filaments were treated as ideal chains without any pair interactions between non-connected beads. The *l*_p_ of the filament is a function of the angle through which the bonds are permitted to move. The filament was approximated as a worm-like chain with a contour length *l*_c_, whose radius of gyration is known as

$$\left\langle R_{g}^{2}\right\rangle =\frac{1}{3}l_{c}l_{p}-l_{p}{}^{2}+\frac{2l_{p}^{3}}{l_{c}}-\frac{2l_{p}^{4}}{l_{c}^{2}}\left(1-e^{-\frac{l_{c}}{l_{p}}}\right)$$

**Fig 1:**
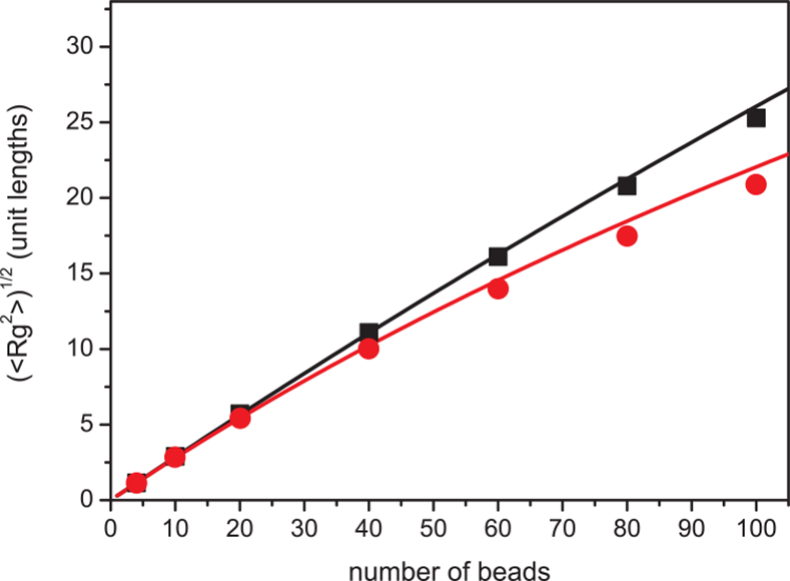
Dependency of the radius of gyration and the filament length for different persistence lengths. Single filaments of various lengths were simulated. After they have been equilibrated 2*10^9^ sweeps were performed. If a maximal bond angel of 15° is allowed (black squares) we found a good agreement with the theoretical prediction for a worm-like chain with a *l*_p_ of 1000 nm (black line). For a bond angle of 25° (red circle) a good comparison with the theoretical prediction for a worm-like chain with a *l*_p_ of 333 nm (red line) was found.

[37]. The range of allowed bond angles was set to a solid angle α = 15° to generate the desired values for the persistence length of vimentin and desmin, *l*_p_ = 1000 nm, and α = 25° for keratins with *l*_p_ = 300 nm (Fig 1) [18,34,38]. The respective persistence length of the three cytoplasmic intermediate filament types has been determined for mature filaments. We do not have indications that the *l*_p_ for unit-length filaments and short filaments is different from that of longer filaments. Therefore and to keep the model simple we assume *l*_p_ to be constant over the whole time scale.

We compared the center of mass diffusion of filaments of different lengths, obtained by MC simulations with previously performed Brownian dynamics simulations [39]. The averaged number of sweeps in the MC simulation, needed by the filament to diffuse over a distance of its own size, is compared to the corresponding time in the Brownian dynamics simulations. This yields an approximate scale for the time unit of an average sweep in the MC simulations of the order of 1ns. Neglecting hydrodynamic interactions leads to some errors in the estimates for the MC time scales: in earlier studies on superhelical DNA dynamics we found that short-range dynamics slowed down by 25% when hydrodynamic interactions were excluded [39].

## Results and Discussion

### Length distributions of vimentin IFs at different stages of assembly

Previous studies had shown that vimentin filament assembly is by and large complete after 1 h under standard conditions, in the sense that filaments are μm in length, ULFs are absent and viscosimetry measurements exhibit a plateau [19]. However, the assembly process continues, though progressively slower: this was directly shown by following the reaction of vimentin filaments, fluorescently labeled with two colors, that were mixed after one hour of assembly and then further incubated for two days [14]. Importantly, back reactions such as the dissociation of tetra- or octameric subunits from mature filaments or breaking of filaments can be neglected [40].

**Fig 2:**
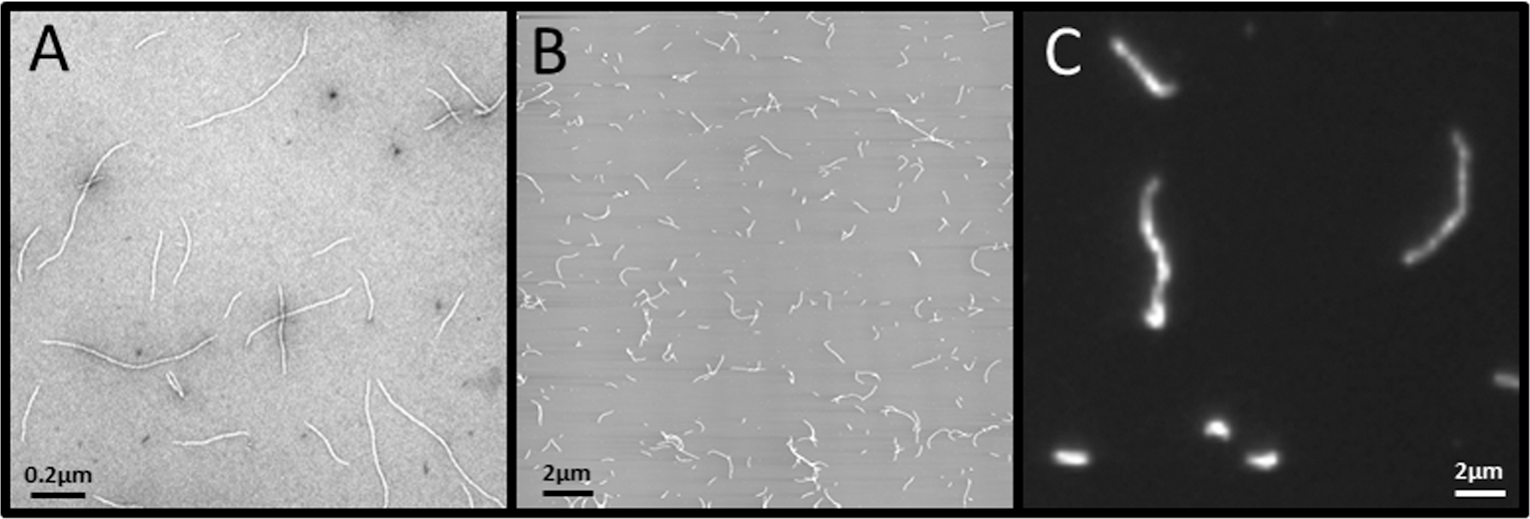
**Comparison of IFs obtained by EM (A) AFM (B) and TIRFM (C).** Assembly was stopped after 10 min for EM and AFM and after 4 h for TIRFM.

To quantitate the assembly process over extended times, we monitored the filament length distribution at 2 h and 4 h after the start of assembly, and as a reference point for the initial phase of assembly we also measured filament length at 10 minutes. At this time point, the filaments are on average less than 1 μm long as illustrated by EM and AFM (Fig 2A and 2B). After 4 h of assembly, TIRFM revealed many very long IFs. Here we show two IFs longer than 6 μm, however, filaments in the 1 μm-range were still present (Fig 2C).

We traced in total almost 9000 filaments, which we estimated to be built from approximately 300,000 ULFs, according to the repeat length of 42.7 nm [24] for an ULF segment within a filament. This 33-fold reduction of the filament number over time reflects “phase II” of the basic IF assembly mechanism, i.e., ULFs and short filaments longitudinally anneal over time to yield longer filaments [12,15,19]. In particular, for the 10 min time point of assembly, 2324 filaments were measured (Table 1). Their mean length *<l>* was 353 nm, corresponding to <*l*_n_> = 7.9 ULF, expressed as the number of ULF-segments within a filament [30]. After 2 h of assembly, 3674 filaments were recorded with a mean length of 1.5 μm or <*l*_n_> = 35 ULF by TIRFM. This value increased to 2.1 μm or <*l*_n_> = 49 ULF after 4 h of assembly, with 2944 filaments measured.

**Table 1:** Assembly kinetics of vimentin.

*t*	*N*	<*l*>	<*l*_*n*_>^a^	SD^b^
10 min	2324	0.353	7.9 (7.4–8.1)	6.2
2 h	3674	1.5	35 (33–36)	32
4 h	2944	2.1	49 (46–51)	45

After assembly time (*t*) the measured numbers of filaments (*N*), the mean length in μm (<*l*>) and the mean length converted to the number of ULFs (<*l*_n_>). The standard deviation (SD) of <*l*_n_> the mean filament length is shown.

^a^ The numbers in brackets show the range of the observed mean lengths from 3 independent experiments.

^b^ The huge standard deviation (SD) is not an experimental error. It does more reflect the broad range of the length distribution of individual filaments at the distinct time points.

**Fig 3:**
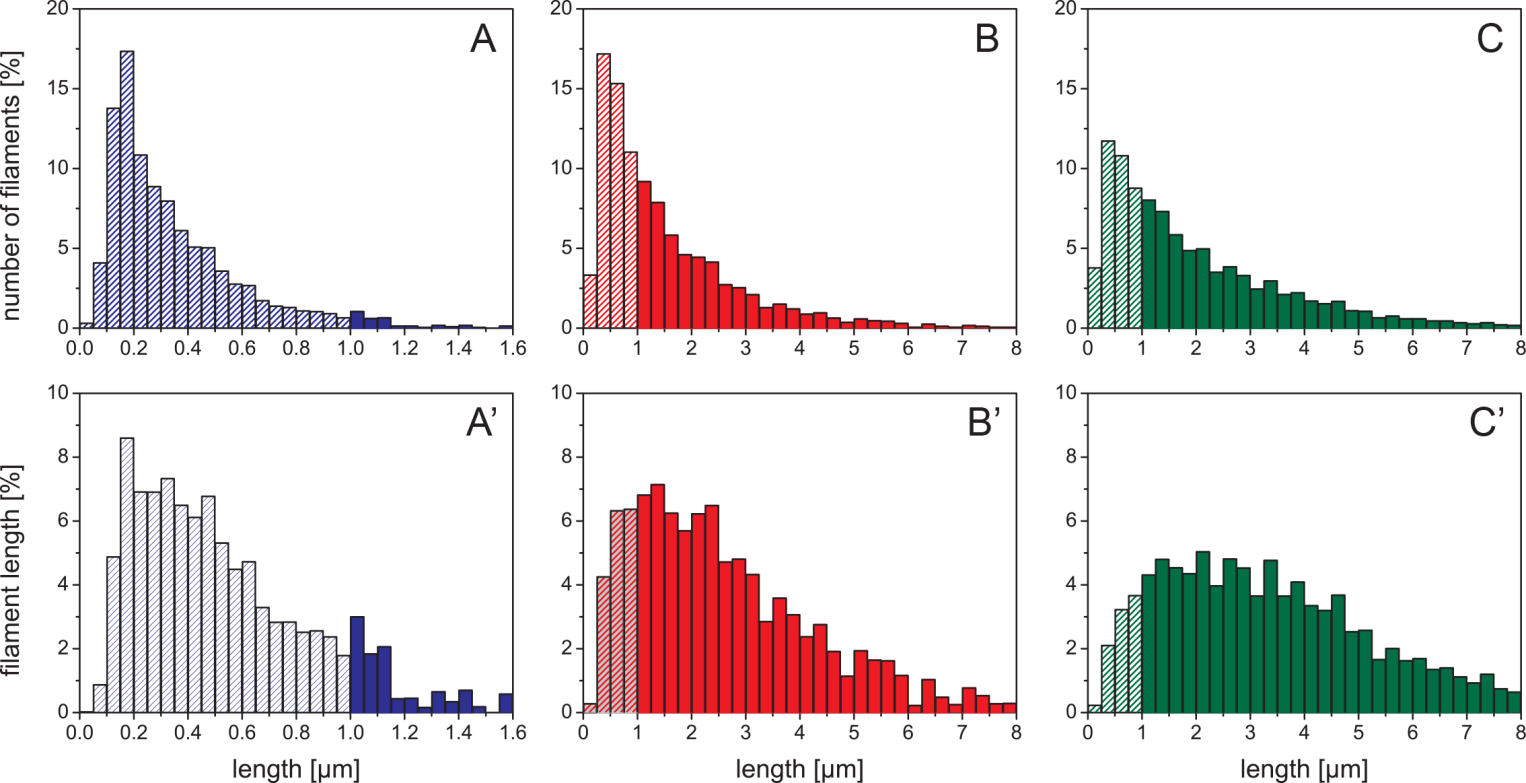
Time-dependent length distribution of vimentin intermediate filaments. Filament length distributions were measured after three assembly times: (A) 10 min by AFM (blue), (B) 2 h (red) and (C) 4 h (green) by TIRFM. Filaments with a measured length less than the persistence length are striped. (A—C) The number of filaments at a certain length is shown at the specific time points. (A’–C’) The proportion of the filament lengths compared to the total filament length observed at a certain length is shown at the specific time points. The size of the bins is set to 1 ULFs (A) and 5 ULFs (B and C).

**Fig 4:**
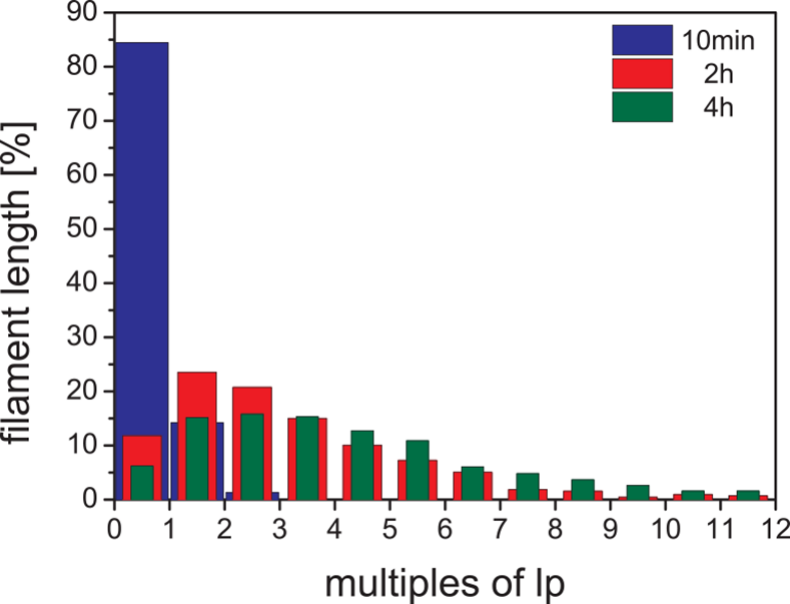
Length distributions of vimentin intermediate filaments normalized to the persistence length. Filament length distributions were measured at three assembly times. The proportion of filaments lengths observed of the indicated lengths is shown at the specific time points.

In addition to the mean length values, the length distributions for the individual time points gives more detailed insight into the filament elongation process: at 10 min only 3% of all filaments are longer than *l*_p_ (Fig 3A). After 2 h of assembly, 56% of the filaments were longer than *l*_p_ (Fig 3B), and 65% after 4 h of assembly (Fig 3C). The effect of the elongation reaction becomes even more evident when one considers the total length of the respective length class. For instance, the 17% of IFs with 0.175–0.200 μm length after 10 min (Fig 3A) represent only 9% of the total filament length (Fig 3A’). After 2h, 17% of total filaments are in the length range from 0.25 to 0.50 μm, corresponding to only 4% of total filament length. Hence, although their number fraction is similar to that of filaments between 0.175 and 0.200 μm at 10 min, their total length represents only half that of the shorter filaments at 10 min. This is due to the fact that after 2 h many longer filaments had formed which contribute little by number but a lot by total polymer length. After 4 h of assembly, the peak of the distribution is much broader compared to that at 10 min and 2 h, as now most of the filament mass is found in filaments with a length between 1 μm and 5 μm. This pronounced shift of total filament length into the longer filament groups is particularly evident when the filament distributions at all three time points are binned with respect to *l*_p_ (Fig 4). After 10 min of assembly, 84 out of 100 ULFs were found in filaments shorter than *l*_p_. At 2 h, this value drops to 12% and at 4 h it is 6%. At both 2 h and 4 h, filaments in the 3 to 4 *l*_p_ range are present with 15%, but filaments longer that 3 to 4 *l*_p_ are 48% at 4 h in comparison to 29% at 2 h. Hence, while the increase of the absolute number of long filaments is not very striking from 2 h to 4 h, the total polymer mass shifts quite significantly to the longer fraction.

Having described filament length over a large cohort of filaments at different time points, the question arose how the progressive decrease of the number of filament ends impacts the kinetics of filament elongation, when most of the filaments are longer than *l*_p_. Moreover we argued that the assembly kinetics should be influenced at some point by the flexibility of the individual chains. In the case of stiff rods the ends scan less space than ends of a flexible chain within the same time period and therefore have less chance to encounter a second end at an appropriate angle.

### Monte Carlo simulations

**Fig 5:**
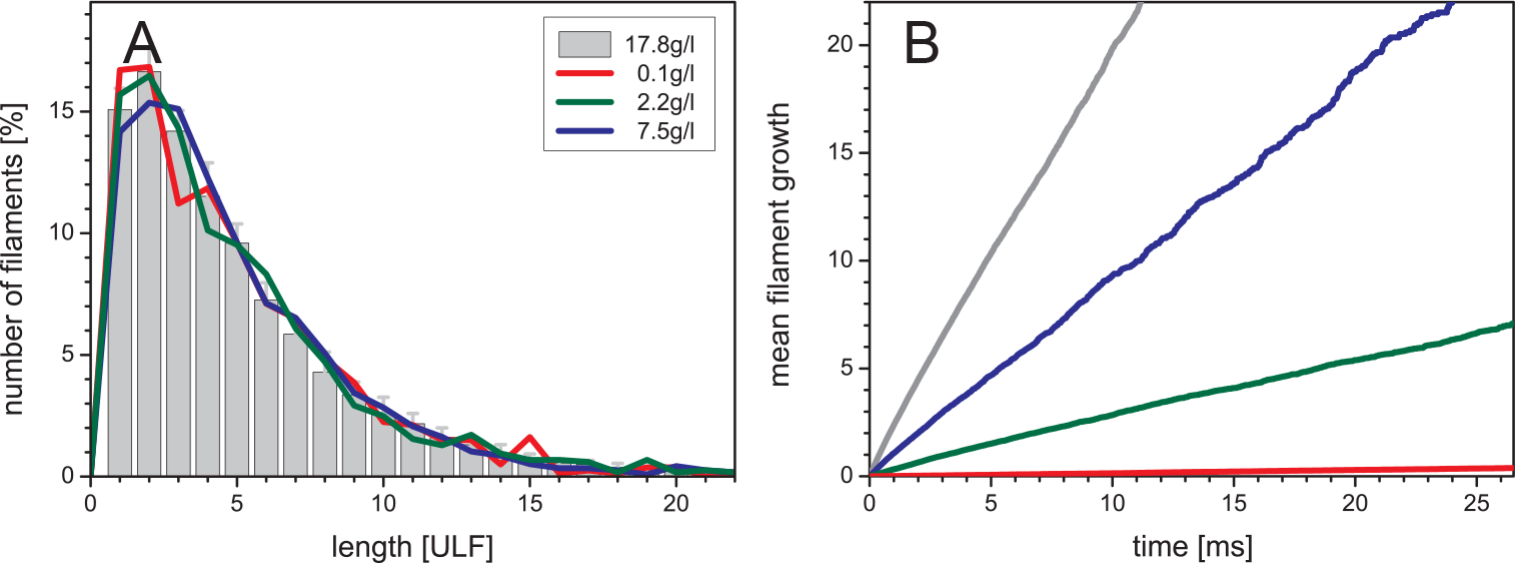
MC simulated filament assembly. At a mean filament length of 5 ULFs length distribution profiles simulated at various concentrations were compared (A). The mean filament growth, <*l*_n_>-1, over time is shown in (B). The volume, the protein concentration, the initial number of ULFs and the number of sweeps to reach the mean length are shown in Table 2. The black line, the grey bars and their standard errors were derived from 10 simulations. Varying the concentration results in comparable length distributions, but the time, or number of sweeps, to reach a given mean length is different.

To reconstruct the experimental assembly process, c_protein_ = 0.1 g/l, we started to simulate the assembly of 1000 ULFs distributed in a cube with an edge length of 3.2 μm. The bond and the reaction solid angles were set to 15° so that the simulated filaments had an *l*_p_ of 1 μm, comparable to the experimental value for vimentin IFs [34,38]. The mean length was <*l*_n_> = 5 after 315 x 10^6^ sweeps (Table 2)., after 750 x 10^6^ sweeps a <*l*_n_> = 10 was reached, and to obtain <*l*_n_> = 22, which corresponds to a value experimentally obtained in the test tube after 2 s, it took 2 x 10^9^ sweeps (1 sweep corresponds roughly to 1ns, see Materials and Methods), corresponding to 11 days of simulations on a standard PC (Intel i7-4770, 8-core 3.4 GHz). In this scenario, the number of filaments went down from 1000 to 45. For a next step, we attempted to simulate filament distributions with <*l*_n_> = 49 such as measured for vimentin at 4 h (Table 1). To reach robust statistical significance for this time point, it was necessary to score a much higher number of filaments. As a consequence, the speed of the simulation had to be increased significantly. Comparing simulated length distributions at <*l*_n_> = 5 at different protein concentrations (Fig 5A), we note that the concentration of filaments did not affect the length distribution, however, the time needed to reach the same <*l*_n_> decreased considerably with increasing protein concentration (Fig 5B). At the high concentration of 17.5 g/l and by allowing for a maximum reaction solid angle of 15°, only 26 x 10^6^ sweeps were needed to reach a <*l*_n_> = 50 with acceptable noise (about 14 days for 4 simulations performed in parallel). In addition, after normalization to 1 g/l, we noted for all concentrations a similar number of sweeps needed to reach a mean filament length of 10 ULFs (Table 2). This result strongly supports the idea that simulations can be performed at significantly higher protein concentrations than used in test tube experiments without altering the specific characteristics of the assembly reaction, and hence this amendment provides a convenient way to lower the simulation time.

**Table 2:** MC simulations of filaments with a *l*_p_ of 1000 nm.

*c* (g/l)^a^	run	*V* (μm^3^)	*n*_0_ [ULF]^b^	sweeps_5_ [10^6^]	sweeps_10_ [10^6^]	*c* ∙ sweeps_10_ [10^6^]^c^	sweeps_50_ [10^6^]
0.10	4	28.0	1000	315 (21)	750 (32)	75	4500^d^
2.19	1	7.63	5832	14.5	33.1	72	--
7.46	1	2.24	5832	4.23	9.63	72	--
17.8	10	0.94	5832	1.77 (0.03)	4.30 (0.13)	77	25.4 (1.0)
17.5	10	7.63	46656	1.74 (0.01)	4.23 (0.03)	74	26.0 (0.6)

Sweeps_n_ is the number of sweeps needed to simulate a <*l*_n_> of 5 ULFs, 10 ULFs and 50 ULFs. The values in brackets are the standard errors of the number of performed simulation runs.

^a^ The equivalent protein concentration in an experiment.

^b^ The initial number of ULFs.

^c^ Number of sweeps normalized to a concentration of 1 g/l. Similar numbers indicate that the elongation reaction is not altered by varying the concentrations in the simulations.

^d^ Estimated value from the proportion sweeps_50_ / sweeps_10_ observed at higher concentrations.

### Simulated length distributions match the experimental data

**Fig 6:**
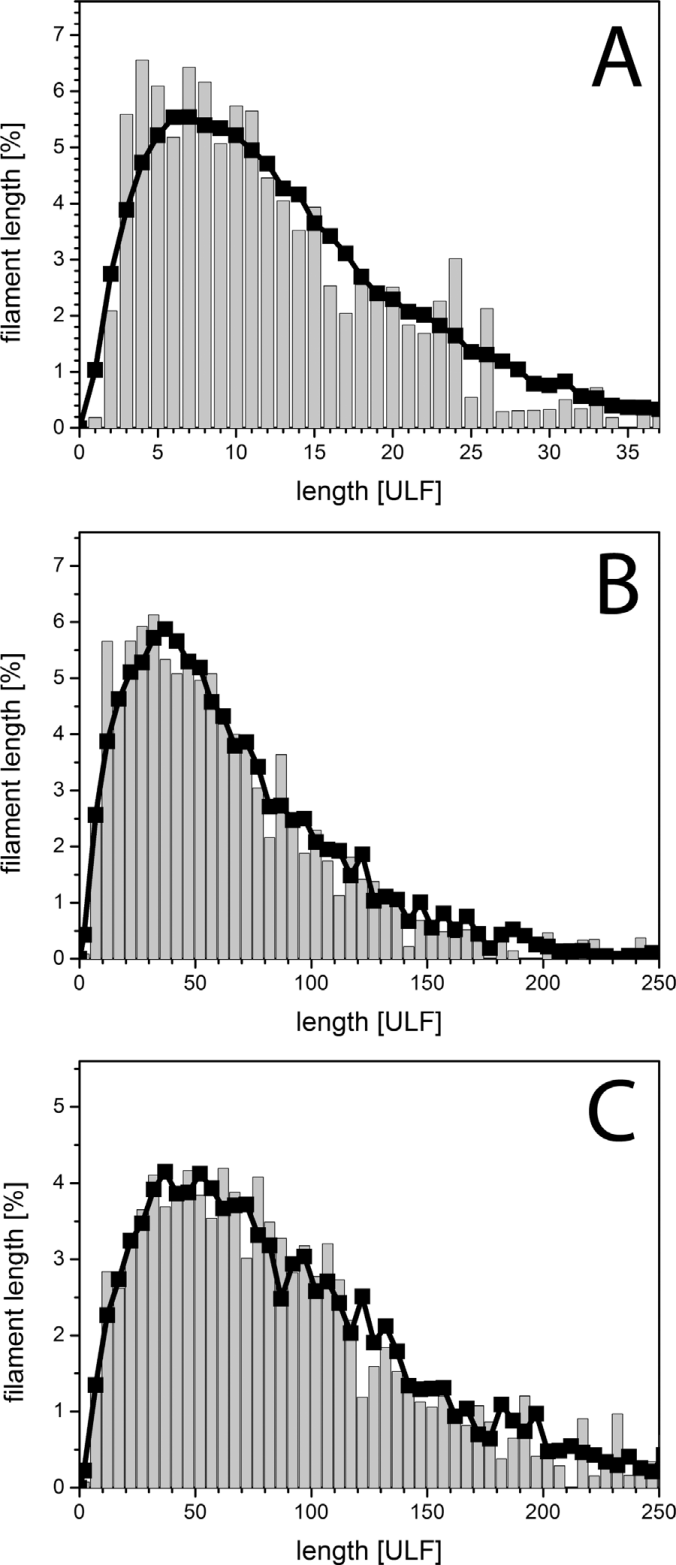
Observed length distributions can be well described by a simple assembly model. Length distribution profiles of vimentin measured at distinct time points are shown (grey bars): A, 10 min; B, 2 h and C, 4 h (see Table 2). To reduce the noise the size of the bins was set to 5 (B,C). The obtained data can be well described by our MC simulations. 10 simulations were performed at protein concentration of 17.5 g/l (for details see bottom Table 2) and mean values at the given <*l*_n_> were calculated (black).

When IF kinetics were modelled under the assumption that filaments behave as stiff rods and associate by end-to-end annealing, calculated filament length distributions for 10 min of assembly fit the experimental data quite well [30]. In contrast, this “stiff rod” model failed to predict filament length profiles measured at 2 h and 4 h [31]. For filaments longer than *l*_*p*_, the flexibility of the filaments has to be considered. In our simulation we now took the flexibility into account, resulting in much faster diffusion and corresponding encounter times of the ends, leading to a broader filament length distribution. These simulated filament length distributions now agree well with the experimental data taken at 10 min, 2 h and 4 h (Fig 6A–6C).

### Assembly kinetics of vimentin, desmin and keratin K8/K18

**Fig 7:**
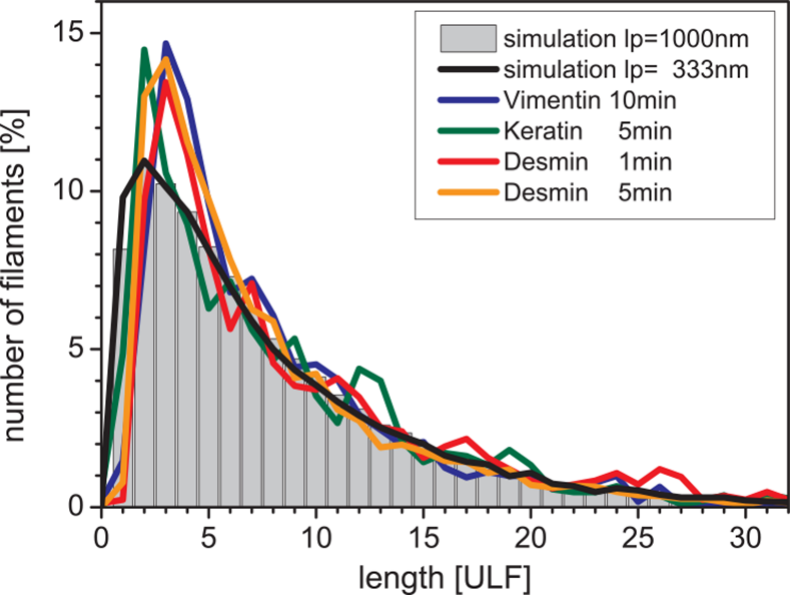
Observed length distributions of cytoplasmic intermediate filaments compared with MC simulations. Mean lengths and assembly conditions are summarized in Table 3 (bold). 10 simulations were performed at protein concentration of 17.5 g/l (grey bars, for details see bottom Table 1). 4 simulations were performed at similar conditions, but the reaction solid angle and the bond angle was set to 25° resulting in a reduction of the persistence length to 1/3 (black line). In both cases the profile at a mean length of 7.9 ULF is shown. Interestingly the shape of the histogram is the same, but the number of sweeps to reach that distribution is decreased by a factor of 4 (Table 3).

In a next step, we compared the assembly of three different IF proteins, i.e. vimentin, desmin and keratin K8/K18. The filament elongation of both desmin and keratin K8/K18 proceeds very similar to that of vimentin. By 2 seconds, ULFs were formed abundantly by lateral aggregation of tetramers; however, their filaments differ with respect to the number of monomers per cross section as measured by scanning transmission electron microscopy (STEM) [32]. In particular, this value ranged from about 16 monomers for keratin K8/K18, to 32 monomers for vimentin and to approximately 48 monomers for desmin. We found different kinetics for the three IFs but the length distribution at a given mean length is comparable (Fig 7). In addition, we established a measure for the kinetics of filament formation, the elongation rate (Table 3, elong. const.), that allows us to directly compare the distinct speeds of filament elongation of different IF proteins assembled under different experimental regimes. In particular, desmin kinetics are comparable to those of vimentin, although keratin K8/K18 assemble about 100 times faster than vimentin. This large difference cannot be explained solely by the difference of the monomers per cross section, which is a factor of 2 and which would result in twice the number of ULFs per mol. Moreover, the lower persistence length of keratin K8/K18 results in a higher reaction solid angle and a higher bond angle, providing a factor of approximately 4 (Table 3). To explain the large difference in assembly speed, we conclude that the intrinsic rate constant *k*_0_ as introduced in Portet et al. [30] is much higher for keratin K8/K18 than for vimentin and desmin. In contrast to keratin K8/K18, both vimentin and desmin have a rather long and complex, intrinsically disordered “pre-coil domain” that may slow down assembly via additional interactions taking place during the head-to-tail overlap association [41]. Nevertheless, as keratins exhibit the identical orientation of their two-stranded coiled coils within tetrameric complexes [18,42] and as the first assembly products are ULFs with identical dimension compared to vimentin and desmin [32], and as furthermore these keratin ULFs do longitudinally anneal with each other to mediate filament growth, we are confident that the same assembly model can be applied.

**Table 3:** Assembly kinetics of cytoplasmic IFs.

IF	*c* [μg/ml]	*t* [s]	<*l*_n_>	elong. const.^a^	*N*	assembly mode^b^
Vimentin^c^	100	300	5.3	0.14	1144	1
Vimentin^c^	100	600	6.9	0.10	839	1
**Vimentin**	**100**	**600**	**7.9**	**0.12**	**2324**	**1**
Vimentin	100	7200	35	0.05	3674	1
Vimentin	100	14400	49	0.03	2944	1
Desmin	20	300	6.3	0.88	6089	1
**Desmin**	**100**	**60**	**9.6**	**1.43**	**833**	**1**
**Desmin**	**100**	**300**	**7.7**	**0.22**	**3691**	**2**
Keratin^**d**^	2.5	60	3.7	18	1049	3
Keratin^**d**^	2.5	120	5.0	13	1066	3
**Keratin**^**d**^	**2.5**	**300**	**8.3**	**9.7**	**3691**	**3**
**MC 15**^**e**^	**17,500**	**0.0043**	**10**	**120**	**4663**	
**MC 25**^**e**^	**17,500**	**0.0010**	**10**	**514**	**4661**	

Values presented in Fig 7 have been highlighted. After assembly time (*t*) the measured numbers of filaments (*N*) and the mean length converted to the number of ULFs (<*l*_n_>) are presented. For the simulations the mean number calculated from different simulations is shown.

^a^ For comparison the observed mean filament lengths are normalized to 1 second assembly at a protein concentration of 1 g/l by (〈*l*_n_〉−1)/(*c*⋅*t*). The Units for the filament type specific elongation constant are [ULF/(g/l)s].

^b^ assembly mode are described in the section Material and Methods

^c^ data taken from [30]

^d^ data were taken from [18]

^e^ simulations were performed see Table 2. The reaction solid angle and the bond angle was set to 15° for filaments with a *lp* of 1000 nm and 25° for the filaments with a *lp* of 333 nm. Data were calculated out of 10 simulations for the stiffer filaments and 4 simulations were performed for the more flexible filaments. The time to reach <*l*_n_> = 10 was estimated from the number of sweeps (see Material and Methods).

## Conclusions

On the basis of the experimental filament length distributions recorded during the assembly of vimentin, desmin and keratin K8/K18, we developed a simulation procedure that allows us to describe the assembly process in the range of minutes to hours by a simple kinetic model for the three types of cytoplasmic IF proteins. As a central element of the model, the filament flexibility serves as a basic parameter for the generation of the Monte-Carlo simulation procedure.

## Abbreviations

AFM: Atomic force microscopy
EM: Electron microscopy
IF: Intermediate filament
MC: Monte Carlo
TIRFM: Total internal reflection fluorescence microscopy
ULF: Unit-length filament
